# Zirconium Molybdate Nanocomposites’ Sensing Platform for the Sensitive and Selective Electrochemical Detection of Adefovir

**DOI:** 10.3390/molecules27186022

**Published:** 2022-09-15

**Authors:** Wenming Li, Jingyun Xiao, Liangyuan Yao, Yanping Wei, Jinsong Zuo, Weili Zeng, Jianhua Ding, Quanguo He

**Affiliations:** 1 Cardiology Department, Zhuzhou People’s Hospital, Zhuzhou 421007, China; 2 School of Life Science and Chemistry, Hunan University of Technology, Zhuzhou 412007, China; 3 Hunan Qianjin Xiangjiang Pharmaceutical Joint Stock Co., Ltd., Zhuzhou 412001, China

**Keywords:** Adefovir, zirconium molybdate, multi-walled carbon nanotubes, electrochemical detection, serum, urine

## Abstract

Adefovir (ADV) is an anti-retroviral drug, which can be used to treat acquired immune deficiency syndrome (AIDS) and chronic hepatitis B (CHB), so its quantitative analysis is of great significance. In this work, zirconium molybdate (ZrMo_2_O_8_) was synthesized by a wet chemical method, and a composite with multi-walled carbon nanotubes (MWCNTs) was made. ZrMo_2_O_8_-MWCNTs composite was dropped onto the surface of a glassy carbon electrode (GCE) to prepare ZrMo_2_O_8_-MWCNTs/GCE, and ZrMo_2_O_8_-MWCNTs/GCE was used in the electrochemical detection of ADV for the first time. The preparation method is fast and simple. The materials were characterized by X-ray powder diffraction (XRD), scanning electron microscopy (SEM), energy dispersive spectroscopy (EDS) and cyclic voltammetry (CV). It was electrochemically analysed by differential pulse voltammetry (DPV). Compared with single-material modified electrodes, ZrMo_2_O_8_-MWCNTs/GCE showed a vastly improved electrochemical response to ADV. Moreover, the sensor complements the study of the electrochemical detection of ADV. Under optimal conditions, the proposed electrochemical method showed a wide linear range (from 1 to 100 μM) and a low detection limit (0.253 μM). It was successfully tested in serum and urine. In addition, the sensor has the advantages of a simple preparation, fast response, good reproducibility and repeatability. It may be helpful in the potential applications of other substances with similar structures.

## 1. Introduction

The chemical name of Adefovir (ADV) is 9-(2-phosphonomethoxyethyl)adenine [1]. ADV has shown positive efficacy in the treatment of various viruses, including HIV, herpes viruses and liver viruses, and has a slower rate of development of resistance than Lamivudine, the first oral drug used to treat HBV [2,3]. ADV is relatively safe, and can be used to treat chronic hepatitis B [4]. ADV was approved for marketing by the Food and Drug Administration (FDA) in 2002 and received European Union (EU) marketing authorisation the following year [5]. ADV is the simplest acyclic nucleoside phosphonate (ANP) analogue. One of the reasons for its low oral bioavailability is the limited intestinal permeability of phosphates. However, its oral prodrug, Adefovir dipivoxil, overcomes this, having good oral bioavailability [6]. The antiviral effect of ANP analogues is based on specific interactions between the active diphosphorylated metabolites and viral DNA polymerase [7]. After entering the body, ADV is phosphorylated by cellular kinases to the active metabolite diphosphate, which then competes with the natural substrate deoxyadenosine triphosphate for viral polymerase incorporation. This incorporation leads to the termination of the viral transcript chain. In order to further understand the intracellular metabolism of Adefovir and other nucleosides and nucleotide analogues, it is necessary to develop a reliable quantitative method [8]. Compared with several other nucleoside analogues, such as Acyclovir and Ganciclovir, ADV bypasses the initial phosphorylation step [9], and is more easily absorbed and utilised by the human body. When ADV is used in long-term treatment, some patients may exhibit drug-related nephrotoxicity [1,10]. Furthermore, it is necessary to study ADV using reliable quantitative methods in clinical metabolic processes. Thus, the quantification of ADV content is essential for people’s health.

At present, a series of methods for detecting ADV have been reported, such as liquid chromatography-tandem mass spectrometry [11], liquid chromatography mass spectrometry [12,13], capillary electrophoresis [14], liquid chromatography [15,16], etc. However, these methods are limited by their long analysis time or complex pre-treatment processes, which limits their use in the detection of substances. Electrochemical methods for the determination of additives, small biological molecules and other materials are advantageous, having flexibility, quickness, environmental friendliness and easy operation [17,18,19,20,21,22,23,24,25,26]. They also allow for the electrochemical behavior of a given drug to be determined through mechanistic studies, and can even determine the interaction mechanisms between the drug and living cells, and the processes that occur in vivo, after the drug has been taken [27]. At present, square wave voltammetry (SWV) [28] and differential pulse voltammetry (DPV) [29] are used for the electrochemical detection of ADV, which demonstrates that ADV exhibits electrochemical activity. Therefore, it is important to find a more suitable material for modification of the electrodes to improve their electrochemical activity. 

Metallic oxide nanomaterials have been used in various ways; for example, tungsten oxide has been explored to detect gases such as NH_3_, NO, HCHO, O_2_ and H_2_S [30], Pd-loaded ZnO has been prepared to detect methane [31], and they have been successfully used as electrode materials [32,33,34,35,36,37,38,39]. Zirconium oxide (ZrO_2_) is a kind of metallic oxide nanomaterial used in various applications, such as in electrochemical sensors [40]. However, it has a band gap width of approximately 5.0 eV [41], compared to a band gap of only about 2.57 or 2.74 eV for zirconium molybdate (ZrMo_2_O_8_). Therefore, ZrMo_2_O_8_ exhibits excellent photocatalytic properties [42,43]. Bimetallic oxide nanoparticles have received significant attention due to their high specific surface area and excellent selectivity [44]. Molybdate is an eco-friendly inorganic corrosion inhibitor [45], and molybdenum shows remarkable properties in the transfer of electrons to electrodes [46]. At present, erbium molybdate [47], nickel molybdate [48], cerium molybdate [49] and other molybdates have been successfully used for electrochemical detection. It has been shown that bimetallic oxides containing zirconium and molybdenum have advantages in catalysis, ion exchange, and solid conductivity [44]. In addition, soluble acid salts of zirconium ions are widely used as inorganic ion exchange materials [50], chemical sensors [51] and so on, due to their excellent properties. ZrMo_2_O_8_ can not only be used as a negative thermal expansion (NTE) material for MX_2_O_8_-type compounds (M = Zr, Ti, Hf and X = P, V, Mo, W) [52,53], but also as a catalyst, and in ion exchangers, luminescent materials, humidity sensors, electrochemical sensors, scintillator materials, energy storage devices and photocatalysts [54,55]. These properties and applications mean that ZrMo_2_O_8_ has attracted a great deal of interest. ZrMo_2_O_8_ has several crystal phases: monoclinic phase (β), trigonal phase (α), cubic phase (γ), orthorhombic phase (LT) and high-pressure phases (monoclinic (δ) and triclinic (ε)). Their conversion is mainly caused by temperature and pressure [56]. The relatively stable crystal phases, under atmospheric pressure, are the α and β crystal phases [43]. 

ZrMo_2_O_8_ can be synthesized by co-precipitation, high-pressure–high-temperature synthesis and non-hydrolytic sol-gel methods [57]. The morphology of ZrMo_2_O_8_ has been reported as spherical [55], rod-shaped [58], etc. Lind and Wilkinson [59] used Zr(OiPr)_4_·iPrOH as a precursor to form ZrMo_2_O_8_ pristine gels, which were later subjected to heat treatment to obtain α-ZrMo_2_O_8_; Shivanekar and Chudasama [60] used zirconyl chloride octahydrate and ammonium molybdate as raw materials to synthesize ZrMo_2_O_8_ for the catalytic decomposition of hydrogen peroxide; Lind et al. [54] obtained γ-ZrMo_2_O_8_ by calcinating zirconium molybdate precursors for the synthesis of cubic-phase zirconium tungstate in the first preparation and characterization of γ-ZrMo_2_O_8_; Lind et al. [58] found that zirconium perchlorate was the most suitable precursor, followed by zirconium nitrate and zirconium chloride, when forming ZrMo_2_O_8_ with a rod-shaped structure; Mancheva et al. [56] prepared ZrMo_2_O_8_ precursors with ZrOCl_2_·2H_2_O and Na_2_MoO_4_·2H_2_O as raw materials using a co-precipitation method, and systematically investigated the phase transition conditions of ZrMo_2_O_8_. 

ZrMo_2_O_8_ also shows advantages in electrochemical detection. Nataraj et al. [61] synthesised ZrMo_2_O_8_@rGO using a simple hydrothermal method and prepared the corresponding electrode to detect hydroquinone with differential pulse voltammetry (DPV), which showed favourable results when detecting in river water, wastewater and tap water. The electrode also had excellent anti-interference properties, stability, repeatability and reproducibility. The construction of hydrogen peroxide electrochemical sensors based on spherical ZrMo_2_O_8_ nanomaterials was first reported by Kumar et al. [55], and the results showed that the ZrMo_2_O_8_ material prepared by a hydrothermal method has a fine crystallinity, regular spherical structure and excellent electrochemical properties. The sensor showed great selectivity even if there were other co-interfering biological substances. Meanwhile, it was shown that ZrMo_2_O_8_ is selective to phosphate, having been successfully used to remove phosphate from wastewater [62,63]. 

Multi-walled carbon nanotubes (MWCNTs) have some excellent properties, such as a high electrical conductivity and high specific surface area. These make them excellent nanomaterials for use in the development of sensor devices [64]. Although MWCNTs have many advantages as carbon materials, they lack selectivity for target molecules when used alone in sensor devices [65]. Therefore, MWCNTs are often combined with other materials to improve their selectivity.

In this work, a simple and fast method was designed based on the idea of compounding, which considers the properties of both ZrMo_2_O_8_ and MWCNTs. ZrMo_2_O_8_ was synthesized using the wet chemical method and MWCNTs were compounded to form ZrMo_2_O_8_-MWCNTs suspension, and then dropped onto the glassy carbon electrode (GCE) to prepare the modified electrode (ZrMo_2_O_8_-MWCNTs/GCE). The electrochemical properties of ZrMo_2_O_8_-MWCNTs/GCE were investigated by cyclic voltammetry (CV) and differential pulse voltammetry (DPV). The sensor has excellent selectivity, repeatability and reproducibility, and low detection limits. Satisfactory results were obtained when ZrMo_2_O_8_-MWCNTs/GCE was used to determine ADV in biological samples.

## 2. Experimental

### 2.1. Experimental Materials

Adefovir (ADV) was purchased from Bidepharm Technology Co., Ltd. (Shanghai, China) (stored at −20 °C); zirconium oxychloride octahydrate, multi-walled carbon nanotubes (MWCNTs), ammonium molybdate tetrahydrate, *N*,*N*-dimethylformamide (DMF), sodium acetate trihydrate, potassium ferricyanide, potassium ferricyanide, potassium chloride and anhydrous ethanol were purchased from Aladdin Biochemical Technology Co., Ltd. (Shanghai, China); glacial acetic acid was purchased from Sinopharm Chemical Reagent Co., Ltd. (Shanghai, China). Other reagents used in the laboratory are analytically pure and were used directly. Serum was provided by the local central hospital (stored at −4 °C) and urine was provided by laboratory staff. The solutions in the experiments were prepared with deionised water (the resistivity being18.2 MΩ).

The preparation method was to weigh 0.0273 g ADV into a 100 mL brown volumetric flask, and then dilute this with deionized water to prepare a 1 mM ADV stock solution, protected from light. A suitable mixture of 1 M acetic acid and 1 M sodium acetate stock solution was used to prepare acetic acid–sodium acetate buffer solution as a support solution for the working solution.

### 2.2. Apparatus

Electrochemical measurements were carried out on a CHI660E electrochemical workstation from Shanghai Chenhua Instruments Co., Ltd. (Shanghai, China). This was achieved using a conventional three-electrode system (with ZrMo_2_O_8_-MWCNTs/GCE as a working electrode, a platinum wire electrode as a counter-electrode and a saturated calomel electrode (SCE) as a reference electrode). The pH of the solution was determined by a digital PHS-3C pH meter from Shanghai Leici Instrument Factory (Shanghai, China). The morphologies of the nanomaterials were characterized by a German Zeiss Sigma 300 scanning electron microscope. The crystal structures of the nanomaterials were characterized using Bruker nano8 Advanced X-ray powder diffraction.

### 2.3. Preparation of ZrMo_2_O_8_ and ZrMo_2_O_8_-MWCNTs Composites

Zirconium oxychloride octahydrate and ammonium molybdate tetrahydrate were weighed to achieve a molar ratio of Zr^4+^ to Mo^6+^ of 1:2, and then dissolved in deionized water. Zirconium oxychloride octahydrate solution was then slowly added dropwise to the ammonium molybdate solution, with constant stirring, to form a white precipitate. Concentrated hydrochloric acid was added to the precipitate until the white precipitate was completely dissolved. This was aged overnight and refluxed at 100 °C for 12 h, at which point the white precipitate was recreated. The white precipitate was washed by centrifugation with water and ethanol several times and baked at 60 °C for 6 h to obtain the zirconium molybdate precursor (ZrMo_2_O_7_(OH)_2_·2H_2_O). This was then calcined at 350 °C for 3 h and, after cooling naturally, the temperature was increased to 400 °C for 2 h to obtain ZrMo_2_O_8_ nanoparticles. Subsequently, 1 mg of ZrMo_2_O_8_ was dissolved in 1 mL of DMF to form its corresponding dispersion, and 1 mg of MWCNTs was dissolved in 1 mL of DMF to form its corresponding dispersion. Both were sonicated for 1 h to obtain homogeneous dispersions. Then, the ZrMo_2_O_8_-MWCNTs composite dispersion was obtained by mixing them with an equal mass ratio.

### 2.4. Fabrication of Modified Electrodes

First, a bare GCE was carefully polished on smooth suede containing 1.0, 0.3 and 0.05 μM Al_2_O_3_ powder in sequence, until the electrode surface was smooth. Then, the GCE was alternately washed with anhydrous ethanol and deionized water. Later, the GCE was dried under an infrared light. A certain amount of ZrMo_2_O_8_-MWCNTs composite dispersion was collected in a pipette gun, dropped onto the surface of GCE and dried under infrared light to obtain ZrMo_2_O_8_-MWCNTs/GCE. The other electrodes (ZrMo_2_O_8_/GCE, MWCNTs/GCE) were prepared similarly.

### 2.5. Electrochemical Detection

**Figure 1:**
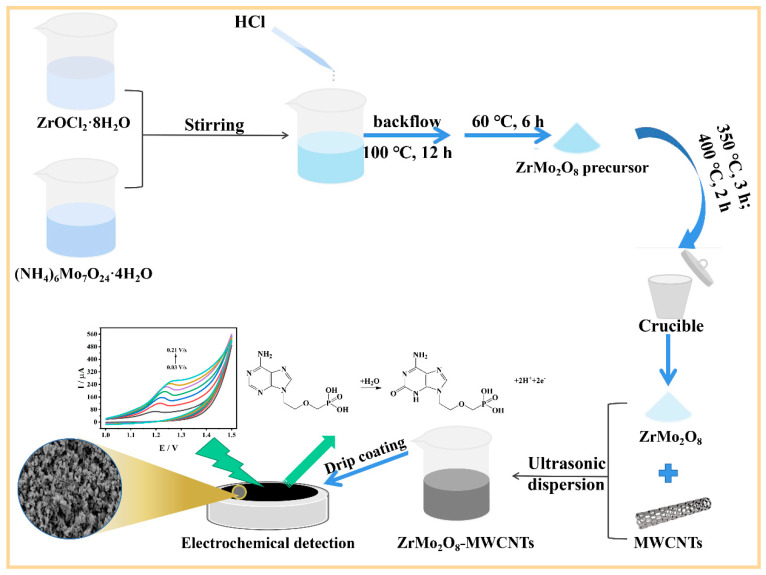
Schematic of the preparation process of ZrMo_2_O_8_-MWCNTs/GCE and its detection of ADV.

Electrochemical detection of ADV was carried out by differential pulse voltammetry (DPV) on ZrMo_2_O_8_-MWCNTs/GCE. A freshly prepared ZrMo_2_O_8_-MWCNTs/GCE was scanned several times in a blank solution by cyclic voltammetry (CV) to activate the electrode before its first use. The electrode was transferred to a working solution of ADV, diluted with a supporting solution, for detection. After the parameters of each measurement condition were achieved, stirring was stopped and the electrochemical workstation was allowed to rest for a while, before continuing to the next parameter. All electrochemical measurements for this work were carried out at room temperature, and the experimental materials and apparatus used in this work were also at room temperature unless otherwise specified. The preparation process for ZrMo_2_O_8_-MWCNTs/GCE and its detection of ADV is shown in Figure 1.

## 3. Results and Discussion

### 3.1. Crystal Phase and Morphology Characterization

**Figure 2:**
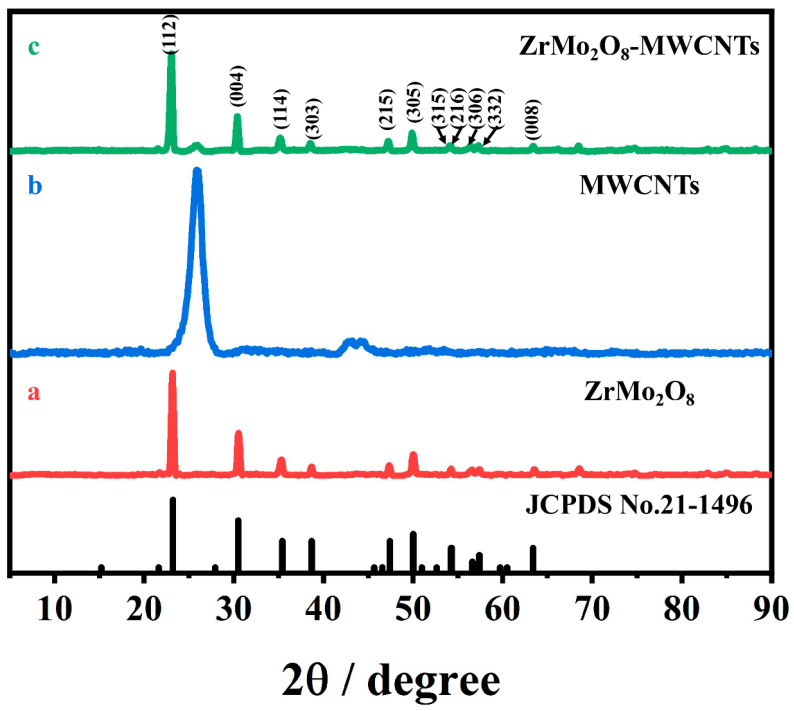
The XRD patterns of ZrMo_2_O_8_ (**a**), MWCNTs (**b**) and ZrMo_2_O_8_-MWCNTs composites (**c**).

The crystal structures of ZrMo_2_O_8_, MWCNTs and ZrMo_2_O_8_-MWCNTs composites were investigated by X-ray powder diffractometry (XRD). As shown in Figure 2, diffraction peaks were observed at 2θ = 23.14°, 30.44°, 35.32°, 38.61°, 47.38°, 49.99°, 54.16°, 54.27°, 56.59°, 57.36° and 63.35°, which correspond to the crystal planes of (112), (004), (114), (303), (215), (305), (315), (216), (306), (332) and (008), respectively. The peak position was consistent with the characteristic diffraction peak of the standard card of ZrMo_2_O_8_ (JCPDS No. 21-1496). The results showed that the synthesized nanomaterial is ZrMo_2_O_8_. In the XRD spectrum of the ZrMo_2_O_8_-MWCNTs composite, the typical diffraction peak in MWCNTs also appeared at 2θ = 26° [66], which was an initial indication that the composite was successfully prepared.

**Figure 3:**
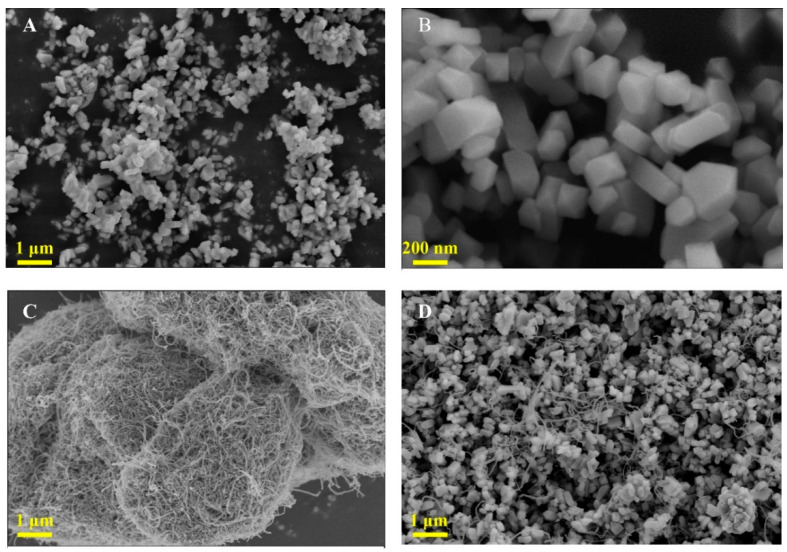
The SEM images of ZrMo_2_O_8_ (**A**,**B**), MWCNTs (**C**) and ZrMo_2_O_8_-MWCNTs composites (**D**).

The morphologies of ZrMo_2_O_8_, MWCNTs and ZrMo_2_O_8_-MWCNTs composites were characterized by scanning electron microscopy (SEM), as shown in Figure 3. Figure 3A,B show the morphologies of ZrMo_2_O_8_ at different magnification sizes; these were observed to be a rod-like structure, which was consistent with the results reported in the literature that the morphology of the ZrMo_2_O_8_ nanomaterial obtained using zirconium oxychloride octahydrate as a precursor is rod-like [58]. The average size of ZrMo_2_O_8_ was approximately 300~400 nm with relatively uniform particles. In Figure 3C, MWCNTs showed an entangled tubular structure. In Figure 3D, the SEM image of ZrMo_2_O_8_-MWCNTs shows that tubular MWCNTs were evenly distributed between ZrMo_2_O_8_ nanorods, and had a stable dispersion state. The detection of the target can be achieved by drop coating ZrMo_2_O_8_-MWCNT composites on the GCE.

**Figure 4:**
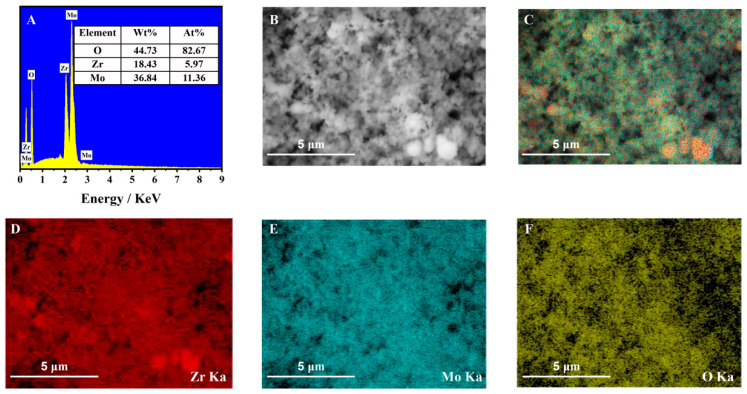
The EDS spectrum of ZrMo_2_O_8_ nanomaterial (**A**); element mappings of all (**C**) and zirconium, molybdenum and oxygen (**D**–**F**) in designated area (**B**) of ZrMo_2_O_8_ nanomaterial.

Figure 4A shows the energy-dispersive spectra (EDS) of the ZrMo_2_O_8_ nanomaterial, in which peaks in oxygen (O), zirconium (Zr) and molybdenum (Mo) can be found. Their contents in the composition were 44.73%, 18.43% and 36.84%, respectively, and their atomic proportions were 82.67%, 5.97% and 11.36%, respectively. The mass ratio and atomic ratio of zirconium and molybdenum to oxygen were compared. The mass ratio of zirconium/oxygen to molybdenum/oxygen in this nanomaterial was approximately 0.5:1, which is larger than the theoretical ratio of 0.47:1 for ZrMo_2_O_8_. The atomic ratio was approximately 0.51:1, which is larger than the theoretical ratio of 0.5:1 for ZrMo_2_O_8_. This may be due to the oxygen in the air and some loss of material. The XRD analysis of ZrMo_2_O_8_ further showed that ZrMo_2_O_8_ nanomaterial was successfully prepared. Figure 4D–F shows the individual elemental mappings of the designated area of the ZrMo_2_O_8_ nanomaterial (Figure 4B), and Figure 4C shows its total elemental mapping. It is clear from the distribution of zirconium, molybdenum and oxygen elements in the ZrMo_2_O_8_ nanomaterial, which were found to be uniformly distributed in the nanomaterial, that the ZrMo_2_O_8_ nanomaterial was successfully prepared.

### 3.2. Electrochemical Characterisation of Different Electrodes

**Figure 5:**
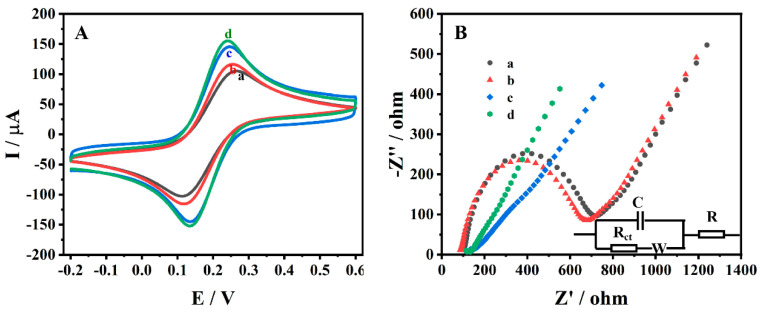
CV diagrams (**A**) and Nyquist plots (**B**) obtained on different electrodes in a solution of 5 mM K_3_[Fe(CN)_6_]^3−/4−^ and 0.1 M KCl (a: GCE, b: ZrMo_2_O_8_/GCE, c: MWCNTs/GCE, d: ZrMo_2_O_8_-MWCNTs/GCE).

GCE, ZrMo_2_O_8_/GCE, MWCNTs/GCE and ZrMo_2_O_8_-MWCNTs/GCE were immersed in 5 mM [Fe(CN)_6_]^3^^−^^/4−^ and 0.1 M KCl, and electrochemically characterized by CV, at a scan rate of 0.1 V/s, in the potential range of −0.2~0.6 V (Figure 5A). They all show a well-defined pair of quasi-reversible peaks. The currents for the oxidation peak (*I_pa_*) and reduction peak (*I_pc_*) on GCE were 111.1 μA and 108.1 μA, respectively, and the corresponding values of potential for oxidation peak (*E_pa_*) and the reduction peak (*E_pc_*) were 0.266 V and 0.112 V, respectively. On ZrMo_2_O_8_/GCE, the values of *I_pa_* and *I_pc_* were 128.4 μA and 126.1 μA, respectively, and the value of ∆*E_p_* (∆*E_p_* = *E_pa_* − *E_pc_*) was 0.137 V. The values of these currents were slightly higher than those of GCE, which may be due to the excellent electrical conductivity of ZrMo_2_O_8_. On MWCNTs/GCE; the values of *I_pa_* and *I_pc_* were 150.1 μA and 151.6 μA, respectively, and the value of ∆*E_p_* was 0.111 V. The peak current of MWCNTs/GCE was higher than that of GCE, which may be attributed to the excellent conductivity and catalytic properties of MWCNTs. Compared with the other electrodes, ZrMo_2_O_8_-MWCNTs/GCE had the highest redox peaks. The values of these currents were 162.4 μA and 164.9 μA, respectively and the ∆*E_p_* was 0.107 V. This may be due to the synergistic effect of ZrMo_2_O_8_ and MWCNTs, the combination of which improves the sensor performance. The surface active area of these electrodes was calculated according to the Randles–Sevcik equation [67]:

(1) $$I_{p}=\left(2.69\times 10^{5}\right)n^{3/2}D^{1/2}v^{1/2}AC$$

In the above formula, *I_p_* is the peak current of K_3_Fe(CN)_6_ (A), n is the number of transmitted electrons, *A* is the effective area (cm^2^), *D* is the diffusion coefficient of K_3_Fe(CN)_6_ (7.6 × 10^−6^ cm^2^s^−1^), *v* is the scan rate (V/S) and *C* is the concentration of K_3_Fe(CN)_6_ (mol/cm^3^). The active surface areas for the four GCE, ZrMo_2_O_8_/GCE, MWCNTs/GCE and ZrMo_2_O_8_-MWCNTs/GCE electrodes were calculated to be 0.09475 cm^2^, 0.1095 cm^2^, 0.1280 cm^2^ and 0.1385 cm^2^, respectively. The results showed that the ZrMo_2_O_8_-MWCNTs composites considerably increased the effective active surface area of the electrode, which had better conductivity and more electroactive sites.

Electrochemical impedance spectroscopy (EIS) was used to obtain more information on charge transfer at the electrode/electrolyte interface. Figure 5B shows the Nyquist plots of GCE (a), ZrMo_2_O_8_/GCE (b), MWCNTs/GCE (c) and ZrMo_2_O_8_-MWCNTs/GCE (d) in 5 mM [Fe(CN)_6_]^3^^−^^/4−^ and 0.1 M KCl, which included a semicircle in the high-frequency region and a straight line in the low-frequency region, reflecting the electron transfer properties and diffusion properties. In the Nyquist plot, the charge transfer resistance (*R_ct_*) is related to the size of the semicircle in the high-frequency region. The Nyquist plots of GCE and ZrMo_2_O_8_/GCE showed two distinct semicircles, and the size of the ZrMo_2_O_8_/GCE semicircle was smaller than that of the GCE, which is related to the better electrical conductivity of ZrMo_2_O_8_. The Nyquist plots of MWCNTs/GCE and ZrMo_2_O_8_-MWCNTs/GCE showed almost straight lines for their curves in the high-frequency area, which indicated that their impedances were considerably lower. The *R_ct_* values for GCE, ZrMo_2_O_8_/GCE, MWCNTs/GCE and ZrMo_2_O_8_-MWCNTs/GCE were 598.9 Ω, 569.4 Ω, 17.63 Ω and 15.9 Ω, respectively. The impedance of ZrMo_2_O_8_-MWCNTs/GCE was the lowest, with this result being consistent with the CV characterization results. This indicated that the combination of ZrMo_2_O_8_ and MWCNTs has a synergistic interaction and is more conducive to the electrochemical reaction.

### 3.3. Electrochemical Behavior of Adefovir on Different Electrodes

**Figure 6:**
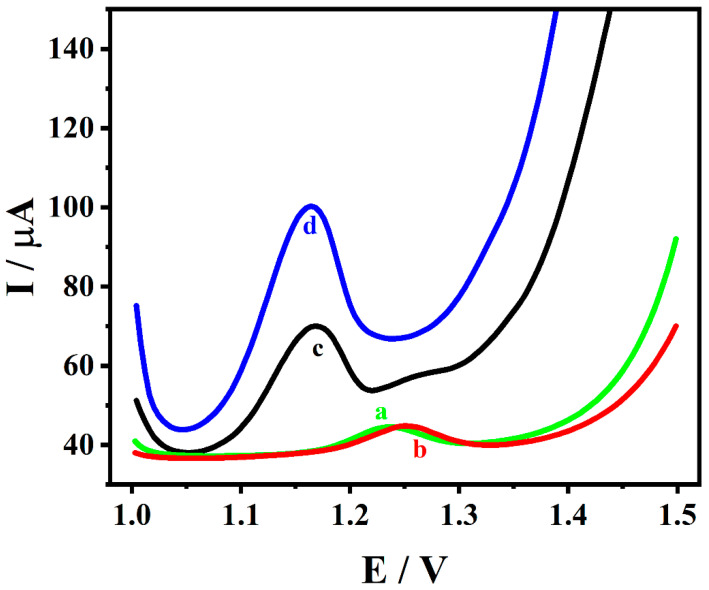
DPV diagrams of 1 × 10^−4^ M ADV on different electrodes (a: GCE, b: ZrMo_2_O_8_/GCE, c: MWCNTs/GCE, d: ZrMo_2_O_8_-MWCNTs/GCE).

The electrochemical performance of GCE (a), ZrMo_2_O_8_/GCE (b), MWCNTs/GCE (c) and ZrMo_2_O_8_-MWCNTs/GCE (d) in 1 × 10^−4^ M ADV solution (acetic acid–sodium acetate buffer solution at pH = 5.5) were investigated by DPV, as shown in Figure 6. It can be seen that the peak current of the ADV was the weakest at the GCE (*I_p_* = 5.149 μA), where the peak potential was 1.236 V. This was lower than the peak current of ZrMo_2_O_8_/GCE, which was found at 1.252 V (*I_p_* = 5.758 μA), indicating that ZrMo_2_O_8_/GCE improved the electrochemical response of ADV to a certain extent. On the MWCNTs/GCE, the peak oxide current of the ADV was increased to 21.35 μA (*E_p_* = 1.168 V), which can be attributed to the excellent conductivity of the MWCNTs. In comparison, the ZrMo_2_O_8_-MWCNTs/GCE obtained a maximum peak current at *E_p_* = 1.164 V, where the peak oxide current of the ADV was 42.57 μA, approximately 8 times higher than that of GCE. This indicates that the synergistic interaction of both ZrMo_2_O_8_ and MWCNTs enhanced the electrocatalytic effect, consequently increasing the electrochemical response of ADV.

### 3.4. Effect of Scan Rate

**Figure 7:**
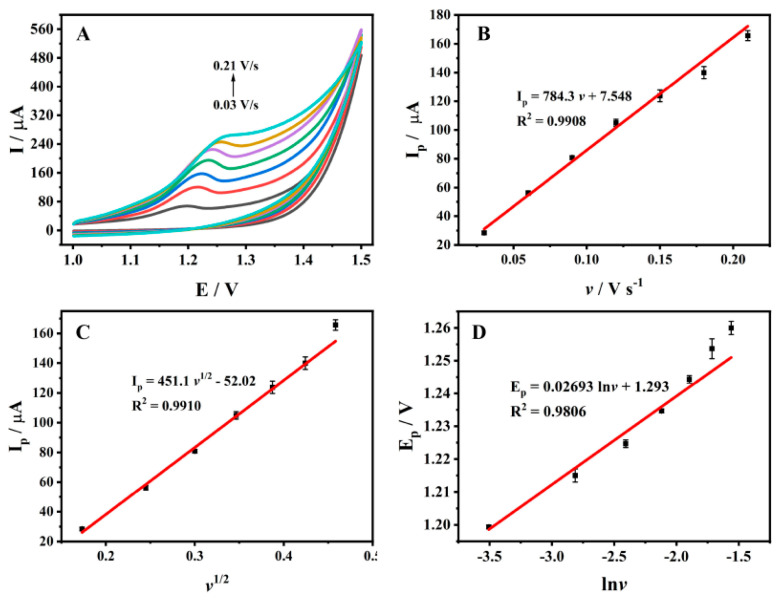
(**A**) CV diagrams of ADV on ZrMo_2_O_8_-MWCNTs/GCE at different scan rates (0.03~0.21 V/s); (**B**) The plot of the peak current versus *v*; (**C**) The plot of the peak current versus *v*^1/2^; (**D**) The plot of the peak potential versus ln*v*.

Under the same conditions, the oxidation peak current and peak potential of 1 × 10^−4^ M ADV on ZrMo_2_O_8_-MWCNTs/GCE were detected by CV by changing the scan rate. Figure 7A shows the CV diagram of ADV at a scan rate range of 0.03~0.21 V/s. It can be seen that the reaction of ADV on ZrMo_2_O_8_-MWCNTs/GCE was an irreversible oxidation reaction. As shown in Figure 7B–C, the linear relationship between peak current and *v* can be expressed as *I_p_* (μA) = 784.3 *v* (V/s) + 7.548 (*R*^2^ = 0.9908), indicating that the oxidation reaction of ADV on ZrMo_2_O_8_-MWCNTs/GCE was controlled by adsorption. The linear relationship between peak current and *v*^1/2^ can be expressed as *I_p_* (μA) = 451.1 *v*^1/2^ (V/s) − 52.02 (*R*^2^ = 0.9910), indicating that the oxidation reaction of ADV on ZrMo_2_O_8_-MWCNTs/GCE was controlled by diffusion. Therefore, the oxidation reaction of ADV on ZrMo_2_O_8_-MWCNTs/GCE was controlled by both adsorption and diffusion. Meanwhile, the peak potential of ADV was linearly proportional to ln*v* (Figure 7D) and its linear regression equation can be expressed as *E_p_* (V) = 0.02693 ln*v* (V/s) + 1.293 (*R*^2^ = 0.9806). For completely irreversible electrode processes, α is usually equal to 0.5 [68]. According to Laviron’s equation [68]:

(2) $$E_{p}=E^{0}+\left(RT/\alpha nF\right)\ln\left(RTk^{0}/\alpha nF\right)+\left(RT/\alpha nF\right)\ln v$$

In the above formula, *E_p_* is the peak potential (V); *E*^0^ is the formal potential (V); *v* is the scan rate (V/s); *α* is the charge transfer coefficient; *k*^0^ is the standard non-homogeneous rate constant; *n* is the number of electron transfers; *T* is the Kelvin temperature (K); *F* is the Faraday constant (96,480 C/mol); and *R* is the molar gas constant (8.314 J/(mol·K)). It can be calculated that *n* = 1.907, approximately equal to 2, meaning that the electrochemical oxidation of ADV on ZrMo_2_O_8_-MWCNTs/GCE was a two-electron reaction.

### 3.5. Effect of pH

**Figure 8:**
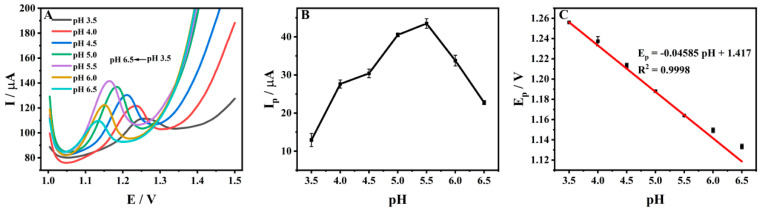
DPV diagrams (**A**), current response (**B**) and the relationship diagram between peak potential and pH (**C**) of ADV on ZrMo_2_O_8_-MWCNTs/GCE at different pH values.

The effect of pH on the acetic acid–sodium acetate buffer solution on the peak current of 1 × 10^−4^ M ADV on ZrMo_2_O_8_-MWCNTs/GCE was investigated by DPV. Figure 8A shows the DPV diagram for 1 × 10^−4^ M ADV in a range of pH values (3.5~6.5), indicating that the strength of the current signal and the position of the peak for ADV on ZrMo_2_O_8_-MWCNTs/GCE was significantly influenced by pH. Taking pH = 5.5 as the dividing point, the peak current first increased and then decreased. When the pH value was 5.5, the oxidation peak current of ADV reached a maximum (Figure 8B). Therefore, pH = 5.5 was chosen for electrochemical detection in subsequent experiments. It can be seen from Figure 7C that the oxidation peak potential negatively shifted with the increase in pH value, and the linear relationship between them can be expressed as: *E_p_* (V) = −0.04585 pH + 1.417 (*R*^2^ = 0.9998). According to the Nernst equation [69]:

(3) $$E^{\theta }=E^{0}-\left(2.303mRT/nF\right)\mathrm{pH}$$

**Figure 9:**
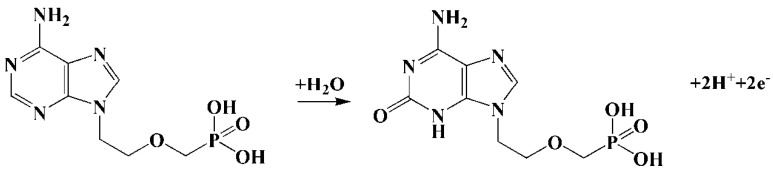
The oxidation mechanism of ADV.

In the above formula, m and n are the number of proton transfers and the number of electron transfers, respectively. The equation slope was −0.04585 and, according to the scanning rate, the oxidation of ADV on ZrMo_2_O_8_-MWCNTs/GCE was a two-electron process. Thus, it can be calculated that the value of m is 1.551, which is approximately equal to 2, indicating that the oxidation of ADV on the ZrMo_2_O_8_-MWCNTs/GCE surface was a two-electron and two-proton reaction. The oxidation mechanism of ADV on ZrMo_2_O_8_-MWCNTs/GCE can be inferred, as shown in Figure 9, which is consistent with the electrochemical oxidation mechanism reported in the literature [29].

### 3.6. Effects of Deposition Conditions and Dropping Amount of ZrMo_2_O_8_-MWCNTs Composites

**Figure 10:**
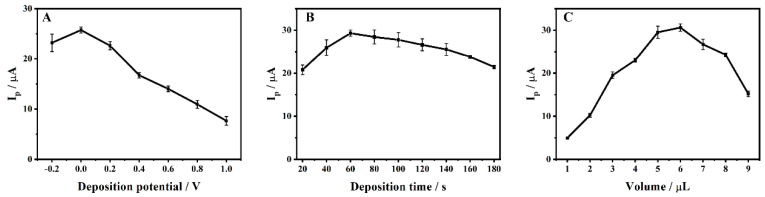
Effects of deposition potential (**A**), deposition time (**B**) and dropping amount (**C**) on oxidation peak current of 1 × 10^−4^ M ADV on ZrMo_2_O_8_-MWCNTs/GCE.

The effects of deposition potential, deposition time and dropping amount of ZrMo_2_O_8_-MWCNTs composites on the electrochemical activity of 1 × 10^−4^ M ADV on ZrMo_2_O_8_-MWCNTs/GCE were investigated by DPV. As shown in Figure 10A, when the deposition potential was −0.2~1.0 V, the degree of ADV adsorption to ZrMo_2_O_8_-MWCNTs/GCE increased. The peak current first increased and then decreased, and reached the maximum at 0 V. At this point, ADV was fully adsorbed. As the deposition potential increased, both the background current and the response current have an effect on the electrochemical detection of the ADV, so 0 V was chosen as the optimal deposition potential. As shown in Figure 10B, the peak current of ADV increased and then decreased with an increasing deposition time of 20~180 s, reaching a maximum at 60 s. This may be due to the fact that the adsorption of ADV on the electrode surface reached supersaturation, resulting in the passivation of ZrMo_2_O_8_-MWCNTs/GCE, which hindered electron transfer and reduced electrode activity. Consequently, 60 s was chosen as the deposition time. The film thickness of the electrode modification material also affects the signal transmission. The effect of different dropping amounts of ZrMo_2_O_8_-MWCNTs composites on the ADV oxidation peak current were investigated in the range of 1~9 μL. As shown in Figure 10C, the peak current increased with the dropping amount in the range of 1~6 μL. When the dropping amount was more than 6 μL, the peak current started to gradually decrease, which may be due to the increase in film thickness hindering electron transfer. For this reason, 6 μL was chosen as the optimal dropping amount. In conclusion, the optimal deposition conditions for the electrochemical detection of ADV on the prepared ZrMo_2_O_8_-MWCNTs/GCE were a deposition potential of 0 V, deposition time of 60 s and dropping amount of 6 μL.

### 3.7. Standard Curve and Detection Limit

**Figure 11:**
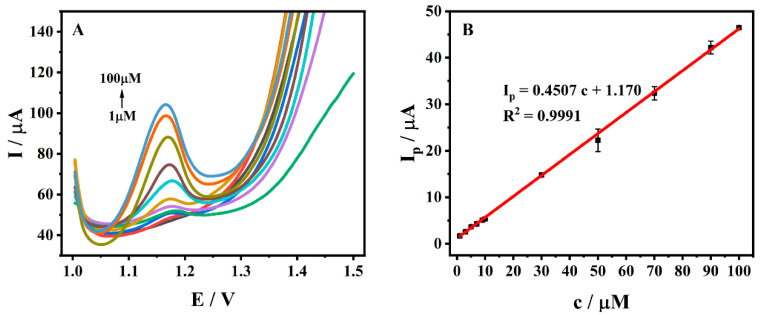
(**A**) DPV diagrams of different concentrations of ADV (1~100 μM); (**B**) The linear relationship between the peak current and ADV concentration in the range of 1~100 μM.

The electrochemical detection of ADV at different concentrations (1~100 μM) was performed by DPV with optimal detection conditions, as shown in Figure 11A. The oxidation peak current of ADV in this range was positively correlated with the magnitude of its concentration (Figure 11B), and the linear regression equation can be expressed as *I_p_* (μA) = 0.4507 *c* (μM) + 1.170 (*R*^2^ = 0.9991). The detection limit was calculated according to the following equation [20]:

(4) LOD=3s/m 

In the above formula, *s* is the standard deviation of the blank signal (*n* = 5), and *m* is the slope of the calibration line within the corresponding range. A detection limit of 0.253 μM (S/N = 3) was obtained. The prepared ADV electrochemical sensor was compared with other detection methods, with the comparison being given in Table 1. By comparison, ZrMo_2_O_8_-MWCNTs/GCE exhibited a wide linear range (1~100 μM) and low detection limit (0.253 μM). Meanwhile, compared with the existing electrochemical methods, the ZrMo_2_O_8_-MWCNTs/GCE prepared in this work has a larger linear range for detecting ADV. The detection limit of this method is lower than that of some traditional methods. Therefore, the ZrMo_2_O_8_-MWCNTs/GCE prepared in this work has advantages, and can be used as an alternative method.

### 3.8. Investigation of Selectivity

**Figure 12:**
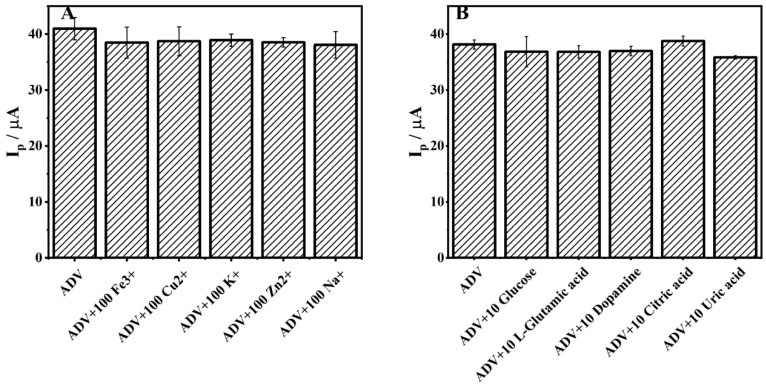
Inorganic (**A**) and organic (**B**) interference experiments of ADV detected by ZrMo_2_O_8_-MWCNTs/GCE.

To investigate the selectivity of ZrMo_2_O_8_-MWCNTs/GCE, potential interfering substances that may be present in real situations were added to the 1 × 10^−4^ M ADV solution under optimal conditions. Three repeated experiments (*n* = 3) were carried out and the average value was taken; the results are shown in Figure 12. Within an allowable relative error of ±5%, 100-fold inorganic ions (Fe^3+^, Cu^2+^, K^+^, Zn^2+^, Na^+^) did not significantly interfere with the electrochemical response of ADV on ZrMo_2_O_8_-MWCNTs/GCE, and neither did 10-fold organic ions (glucose, L-glutamic acid, dopamine, citric acid, uric acid). The results showed that the prepared ZrMo_2_O_8_-MWCNTs/GCE has excellent selectivity for the analysis of ADV, which makes the detection of ADV in real samples feasible, and can be applied to the detection and analysis of complicated samples.

### 3.9. Repeatability, Reproducibility and Stability

**Figure 13:**
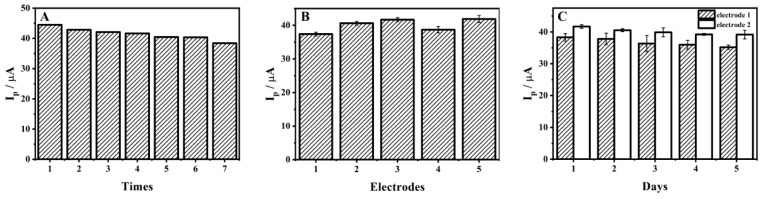
Repeatability (**A**), reproducibility (**B**) and stability (**C**) of ZrMo_2_O_8_-MWCNTs/GCE.

The repeatability, reproducibility and stability of ZrMo_2_O_8_-MWCNTs/GCE were investigated by DPV in a working solution containing 1 × 10^−4^ M ADV to provide a basis for the credibility of the test method. On the same ZrMo_2_O_8_-MWCNTs/GCE, the peak current of 1 × 10^−4^ M ADV in pH 5.5 acetic acid–sodium acetate buffer solution was recorded seven times (Figure 13A) and the relative standard deviation (RSD) of the measured signal was 4.74%, indicating that the sensor has excellent repeatability in the determination of ADV. In addition, five different electrodes were prepared using the same method to investigate the reproducibility of ZrMo_2_O_8_-MWCNTs/GCE. Three repeated experiments (*n* = 3) were carried out and the average value was taken, the results of which are given in Figure 13B. The RSD of the measured signal was 4.88%, indicating that the sensor has an appreciable reproducibility in ADV determination. Two electrodes were prepared, both electrodes were used continuously for five days, and three repeated experiments (*n* = 3) were carried out and the average value was taken. The peak currents of ZrMo_2_O_8_-MWCNTs/GCE were found to be retained at 91.88% and 93.91%, respectively (Figure 13C), indicating that the ZrMo_2_O_8_-MWCNTs/GCE has excellent stability.

### 3.10. Real Sample Detection

To investigate the feasibility of using ZrMo_2_O_8_-MWCNTs/GCE in the determination of ADV in real situations, human serum and urine were used as real samples and tested using method of standard addition and recovery. A total of 1 mL of serum and urine were diluted 100 times, respectively with pH 5.5 acetic acid–sodium acetate buffer solution. Different concentrations of ADV were added and the real samples were detected by DPV under optimal conditions. The results are given in Table 2, with recoveries ranging from 96.05% to 100.0% for serum and 95.86% to 103.8% for urine. This indicated the feasibility of ZrMo_2_O_8_-MWCNTs/GCE for the detection of ADV in real samples.

## 4. Conclusions

In this work, ZrMo_2_O_8_ was synthesized using a wet chemistry method with zirconyl chloride octahydrate as a precursor and used as a nanomaterial to investigate a novel electrochemical method for the determination of ADV by ZrMo_2_O_8_-MWCNTs/GCE. The optimal ZrMo_2_O_8_-MWCNTs/GCE conditions for detecting ADV were determined by optimizing the deposition conditions, dropping amount and pH. The prepared ZrMo_2_O_8_-MWCNTs composites were characterized by XRD, SEM and EDS to determine their crystal phase and morphology characteristics. The results show that the ZrMo_2_O_8_-MWCNTs composites exhibited excellent electrochemical activity for ADV under optimal experimental conditions, with a fine linear correlation in the concentration range of 1~100 μM and a detection limit of up to 0.253 μM. In addition, ZrMo_2_O_8_-MWCNTs/GCE showed favourable selectivity, repeatability, reproducibility and stability for ADV, which enabled satisfactory recoveries to be obtained. This allowed it to be used in the determination of ADV in real serum and urine samples, with satisfactory recovery. The electrochemical sensor was simple to prepare and allows for the easy and quick detection of ADV.

## Figures and Tables

**Table 1:** Comparison of different ADV determination methods.

Method	Material	Linear Range (μM)	LOD (μM)	References
capillary electrophoresis	hydroxypropyl methyl cellulose	0.0366~1.83	0.00977	[14]
liquid chromatography	/	0.366~18.3	0.366	[15]
liquid chromatography mass spectrometry	/	0.0458~0.586 (Serum)	0.00366 (Serum)	[12]
	/	0.000183~0.0293 (Urine)	/	[12]
SWV	^a^ HMDE	1.830~18.30 (Plasma)	0.6956 (Plasma)	[28]
	HMDE	0.9151~8.236 (drug)	0.2928 (drug)	[28]
DPV	^b^ PPy/GCE	0.25~50	0.0031	[29]
DPV	ZrMo_2_O_8_-MWCNTs/GCE	1~100	0.253	this work

^a^ HMDE: Hanging mercury drop electrode. ^b^ PPy/GCE: polypyrrole/glassy carbon electrode.

**Table 2:** Recovery of ADV in actual samples.

Sample	Measured Value (μM)	Amount Added (μM)	Total Determination (μM)	RSD (%)	Recovery Rate (%)
Serum 1	^a^ ND	5	4.908	2.98	98.16
	ND	10	9.996	3.08	99.96
	ND	15	15.00	3.43	100.0
Serum 2	ND	5	4.835	4.83	96.70
	ND	10	9.605	5.03	96.05
	ND	15	14.56	4.36	97.07
Urine 1	ND	5	4.793	5.32	95.86
	ND	10	9.787	1.60	97.87
	ND	15	15.35	1.40	102.3
Urine 2	ND	5	4.884	2.93	97.68
	ND	10	10.38	1.26	103.8
	ND	15	14.87	1.85	99.13

^a^ ND = Not detected.

## Data Availability

Not applicable.

## Abbreviations

ADV: Adefovir; AIDS, acquired immune deficiency syndrome; CHB, chronic hepatitis B; ZrMo_2_O_8_, zirconium molybdate; MWCNTs, multi-walled carbon nanotubes; GCE, glassy carbon electrode; XRD, X-ray powder diffraction; SEM, scanning electron microscopy; EDS, energy dispersive spectroscopy; CV, cyclic voltammetry; DPV, differential pulse voltammetry; ANP, acyclic nucleoside phosphonate; SWV, square wave voltammetry; ZrO_2_, zirconium oxide; NTE, negative thermal expansion; DMF, *N*,*N*-dimethylformamide; SCE, saturated calomel electrode; EIS, electrochemical impedance spectroscopy; HMDE, hanging mercury drop electrode; PPy/GCE: polypyrrole/glassy carbon electrode; RSD, relative standard deviation.
